# A Prognostic DNA Damage Repair Genes Signature and Its Impact on Immune Cell Infiltration in Glioma

**DOI:** 10.3389/fonc.2021.682932

**Published:** 2021-05-28

**Authors:** Guohui Wang, Huandi Zhou, Lei Tian, Tianfang Yan, Xuetao Han, Pengyu Chen, Haonan Li, Wenyan Wang, Zhiqing Xiao, Liubing Hou, Xiaoying Xue

**Affiliations:** ^1^ Department of Radiotherapy, The Second Hospital of Hebei Medical University, Shijiazhuang, China; ^2^ Department of Radiation Oncology, Peking University China-Japan Friendship School of Clinical Medicine, Beijing, China; ^3^ Department of Central Laboratory, The Second Hospital of Hebei Medical University, Shijiazhuang, China; ^4^ Department of Neurological Diagnosis and Restoration, Osaka University Graduate School of Medicine, Suita, Japan; ^5^ Department of Neurosurgery, Third Affiliated Hospital, Southern Medical University, Guangzhou, China

**Keywords:** glioma, DNA repair, tumor microenvironment, prognosis, immune cells

## Abstract

**Objective:**

Glioma is the most frequent type of malignant cerebral tumors. DNA damage repair genes (DDRGs) play a crucial role in the development of cancer. In this study, we constructed a DDRGs signature and investigated the potential mechanisms involved in this disease.

**Methods:**

RNA sequence data, microarray data, and corresponding clinical information of gliomas were downloaded from The Cancer Genome Atlas (TCGA), Chinese Glioma Genome Atlas (CGGA), and Gene Expression Omnibus (GEO). Subsequently, we identified candidate genes by differential analysis and Cox regression analysis. The least absolute shrinkage and selection operator Cox regression model was utilized to construct a DDRGs signature using TCGA training dataset. According to this signature, patients with glioma were divided into low- and high-risk groups. The predictive ability of the signature was validated by prognostic analysis, receiver operating characteristic curves, principal component analysis, and stratification analysis in TCGA testing and CGGA verification datasets. CIBERSORT and single-sample gene set enrichment analysis (ssGSEA) were used to evaluate the immune microenvironment of glioma. Moreover, we conducted GSEA to determine the functions and pathways in the low- and high-risk groups. Finally, a nomogram was constructed by combining the signature and other clinical features.

**Results:**

A total of 1,431 samples of glioma (592 from TCGA, 686 from the CGGA, and 153 from the GEO) and 23 samples of normal brain tissue from the GEO were analyzed in this study. There were 51 prognostic differentially expressed DDRGs. Additionally, five DDRGs (CDK4、HMGB2、WEE1、SMC3 and GADD45G) were selected to construct a DDRGs signature for glioma, stratifying patients into low- and high-risk groups. The survival analysis showed that the DDRGs signature could differentiate the outcome of the low- and high-risk groups, showing that high-risk gliomas were associated with shorter overall survival. The immune microenvironment analysis revealed that more immunosuppressive cells, such as tumor associated macrophages and regulatory T cells, were recruited in the high-risk group. GSEA also showed that high-risk glioma was correlated with the immune and extracellular matrix pathways.

**Conclusion:**

The five DDRGs signature and its impact on the infiltration of immunosuppressive cells could precisely predict the prognosis and provide guidance on the treatment of glioma.

## Introduction

Glioma is the most common type of primary tumors of the central nervous system (CNS), accounting for approximately 70% of cases. It is also a major cause of death among patients with intracranial tumors (1). Patients with glioma are primarily treated with surgical resection, radiotherapy, chemotherapy, and a combination of different therapies. The National Comprehensive Cancer Network guideline recommends chemoradiation with or without tumor-treating fields (TTF) for the adjuvant treatment of primary glioblastoma (GBM). However, the prognosis of GBM remains poor, mainly due to the high risk of recurrence and resistance to chemoradiotherapy (2–4). In addition, gliomas do not have clear boundaries and are characterized by high degrees of infiltration *via* diffusion and a high proliferation rate, thereby complicating surgical resection (5). Recently, some new therapies have been proposed for numerous types of cancer, including immunotherapy and molecular targeted therapy. Randomized controlled clinical studies have shown that these treatments could significantly improve the survival of patients with lung cancer, colorectal cancer, and numerous other types of tumors (6–8). However, some clinical trials have found that these therapeutic modalities are not as effective in patients with glioma. Some researchers argued that this may be attributed to differences in the microenvironments of gliomas and other types of cancer (9).

Radiotherapy and chemotherapy can cause DNA double-strand breaks in tumor cells, which in turn trigger cell apoptosis and death. The DNA damage repair mechanism of tumor cells is abnormally activated; it mainly repairs damaged DNA by homologous recombination and non-homologous end joining. Following DNA double-strand breakage, DNA damage receptors are activated and damage repair proteins, such as BRCA1 DNA repair associated (BRCA1), BRCA2, and RAD51 recombinase (RAD51), are recruited at the damaged sites to repair damaged DNA this process leads to complete resistance to the tumoricidal effect of chemoradiotherapy (10). The alkylating agent temozolomide (TMZ), which can induce DNA breaks in glioma cells and subsequently lead to cell death, is one of the major therapeutic approaches used in patients with glioma. O-6-methylguanine-DNA methyltransferase (MGMT) encodes a DNA damage repair protein that protects tumor cells against DNA double-strand breaks caused by alkylating agents, such as TMZ. Some researchers have demonstrated that the expression levels of MGMT could predict sensitivity to TMZ in patients with glioma (11). Therefore, DNA damage repair genes (DDRGs) play an important role in tumor resistance to chemoradiotherapy.

DNA repair deficiency is also an emerging biomarker of response to immune checkpoint blockade (12). Alterations in DDRGs are associated with genomic instability and increased somatic tumor mutational burden, which in turn promote the generation of tumor-specific neoantigens. The continuous stimulation of the body by tumor antigens generates a persistent immune activation response. This effect depletes or remodels the related effector cells in the tumor microenvironment, thereby impairing their normal functions. In turn, an immunosuppressive microenvironment is generated that promotes tumorigenesis and progression (13). Thus, the tumor microenvironment contains more neoantigens and the function of immune cells of the tumor microenvironment becomes more complex, especially with regards to immunosuppressive cell infiltration. At present, there are few studies on the interaction between DDRGs and the tumor microenvironment of gliomas.

In this study, we collected RNA sequencing data, microarray data, and corresponding clinical information of gliomas from The Cancer Genome Atlas (TCGA), Chinese Glioma Genome Atlas (CGGA), and Gene Expression Omnibus (GEO) databases. Next, we identified survival-related DDRGs by univariate regression analysis. Subsequently, a DDRGs signature for prognostic prediction was constructed using least absolute shrinkage and selection operator (LASSO) regression and Cox proportional hazards regression. The DDRGs signature composed of CDK4, HMBG2, WEE1, SMC3 and GADD45G could accurately predict the prognosis of gliomas. More importantly, we found that the DDRGs signature was strongly associated with immunosuppressive cell infiltration in the microenvironment of gliomas. Our results demonstrated that the DDRGs signature was closely related to prognosis and provided reliable clues for elucidating the microenvironment of glioma.

## Materials and Methods

### Data Acquisition and Identification of Candidate Genes

RNA sequencing data and corresponding clinical information from TCGA LGG and GBM datasets were downloaded from TCGA data portal (https://portal.gdc.cancer.gov/) updated to July 19, 2019. Gene expression profiling and corresponding clinical features of gliomas were obtained from the CGGA database (http://www.cgga.org.cn/) up to May 6, 2020, including the datasets mRNAseq-693 and mRNAseq-325. The microarray dataset GSE4290 for differential gene analysis was downloaded from the GEO database (https://www.ncbi.nlm.nih.gov/geo). RNA sequencing data from TCGA (LGG and GBM datasets) and CGGA (693 and 325 datasets) were normalized and batched using the limma R package. All data were screened to remove samples with missing clinical information. The clinicopathological characteristics of patients in this study are presented in **Table 1**. A total of 513 DDRGs were retrieved from the molecular signature database (MSigDB) (https://www.gsea-msigdb.org/gsea/msigdb) and previous literature (14) (**Supplementary Table S1**). Differentially expressed DDRGs between gliomas and normal brain tissues the GSE4290 dataset were identified using the limma R package and the following criteria: |logFC|>1; false discovery rate <0.05. Next, data from TCGA were randomly divided into the training and testing datasets. Candidate genes in TCGA training dataset were identified through univariate Cox analysis with cut-off values of P<0.001. An interaction network of candidate genes was constructed using the Search Tool for the Retrieval of Interacting Genes (STRING) database (version 11.0) (15).

**Table 1:** Clinicopathological characteristics of glioma patients from the TCGA, CGGA and GEO databases.

	TCGA-Training cohort	TCGA-Testing cohort	CGGA validation cohort	GSE4290
	N = 294	N = 298	N = 686	N = 176
Age				
<42	114	129	307	NA
≥42	180	169	379	NA
Gender				
Male	173	171	399	NA
Female	121	127	287	NA
Normal Tissue	NA	NA	NA	23
Grade				
II	101	110	177	45
III	116	122	226	31
IV	77	66	283	77
IDH				
Wild	116	104	315	NA
Mutation	178	194	371	NA
1p/19q				
Codel	69	80	141	NA
Non-codel	225	218	545	NA
MGMT				
Methylated	NA	NA	386	NA
un-methylated	NA	NA	300	NA
Status				
Dead	91	82	457	NA
Alive	203	216	229	NA
RiskScore				
Low	141	151	343	NA
High	153	147	343	NA

### Construction and Validation of a DDRGs Signature

Candidate genes were analyzed using LASSO regression and Cox proportional hazards regression. The independent variable in the regression was the expression of candidate DDRGs, and the response variable was the prognosis of patients in the training set. Subsequently, the DDRGs signature was constructed by a linear combination of the regression coefficient multiplied by its mRNA expression level:

$$riskScore=\sum_{j=1}^{n}\left(Coefj*Xj\right)$$

Using the formula shown above, we can calculate the risk score for each patient with glioma. Patients were separated into high- and low-risk groups according to the median score. Prognostic analysis was performed with the survminer package in R.

Principal component analysis (PCA) was performed to explore the distribution in the low- and high-risk groups using the Rtsne package, an established dimensionality reduction method (16). Using the survivalROC R package, we constructed a time-dependent receiver operating characteristic (ROC) curve and Harrell’s concordance index to assess the predictive value of the DDRGs signature for prognosis. The online database Gene Expression Profiling Interactive Analysis (GEPIA; http://gepia.cancer-pku.cn/index.html) (17) was used to analyze the expression and prognosis of genes constituting the DDRGs signature in glioma. In addition, the protein expression levels of these genes were determined using the Human Protein Atlas (http://www.proteinatlas.org) online database (18).

### Internal and External Validation of the DDRGs Signature

TCGA testing and CGGA datasets were used for internal and external validation, and a risk score could be calculated for each patient with glioma using the formula shown above. Based on the median values, patients were divided into high- and low-risk groups. Next, the prognostic analysis, PCA, and construction of the time-dependent ROC curve of each patient were performed using R. Univariate and Multivariate Cox regression analyses were used to identify independent prognostic factors.

### Survival Analysis of the DDRGs Signature in Stratified Patients With Glioma

In this study, patients with glioma were stratified according to their clinicopathological features including age, sex, primary-recurrent-secondary (PRS) type, grade, IDH, 1p19q, and MGMT status. The Kaplan–Meier survival analysis was implemented to calculate the survival rates in the low- and high-risk groups of stratified patients.

### Construction of a Predictive Nomogram

In clinical research, nomograms are widely used to predict the outcomes of patients with cancer (19). In the CGGA cohort, all clinical features were utilized for the construction of a nomogram to investigate the probability of 1-, 2-, and 3-year overall survival (OS) of patients with glioma using the rms R package. A calibration curve was used to compare the predictions of this nomogram with the actual rates using the rms package. Specifically, the C-index was used as a discrimination measure considering the censored data in this survival analysis study. To demonstrate the incremental value of the DDRGs signature over the clinicopathological characteristic for individualized assessment of OS, the decision curve was constructed.

### Evaluation of the Glioma Microenvironment Through CIBERSORT and Single-Sample Gene Set Enrichment Analysis (ssGSEA)

CIBERSORT (20) utilizes a deconvolution algorithm to predict the percentages of 22 phenotypes of immune cells in tumor tissue. This method was used to estimate the population fractions of immunocytes in the low- and high-risk groups. Violin plots were generated using the vioplot package to show differences in the infiltration of immunocytes between the high- and low-risk groups. In addition, the degree of immune cell infiltration was quantified using enrichment scores calculated through ssGSEA of the Gene Set Variation Analysis package of the R software. This analysis yielded 29 immune infiltration-related information, including immune cell species, immune function, and immune-related pathways. The patients of glioma were hierarchically clustered to high, medium or low immune group based on ssGSEA scores for those 29 immune infiltration related information.

### Gene Set Enrichment Analyses

Finally, we searched for the underlying molecular mechanism through which the DDRGs signature indicated worse prognosis in patients with glioma using the GSEA 4.0.2 software (21). We used the MsigDB, c2.cp.kegg.v7.2.symbols.gmt and c5.go.bp.v7.2.symbols.gmt as the functional gene sets. Default weighted enrichment statistics were used, and the number of random combinations was set to 1,000. A normalized enrichment score (NES) >1 and false discovery rate <0.05 denoted statistical significance.

### Statistical Analysis

The R software (4.0.0) with corresponding packages and GraphPad Prism 7 (GraphPad Software Inc., San Diego, CA, USA) were used for statistical analyses. Unless specified otherwise above, P<0.05 denoted statistically significant differences.

## Results

### Characteristics of Patients With Glioma

**Figure 1:**
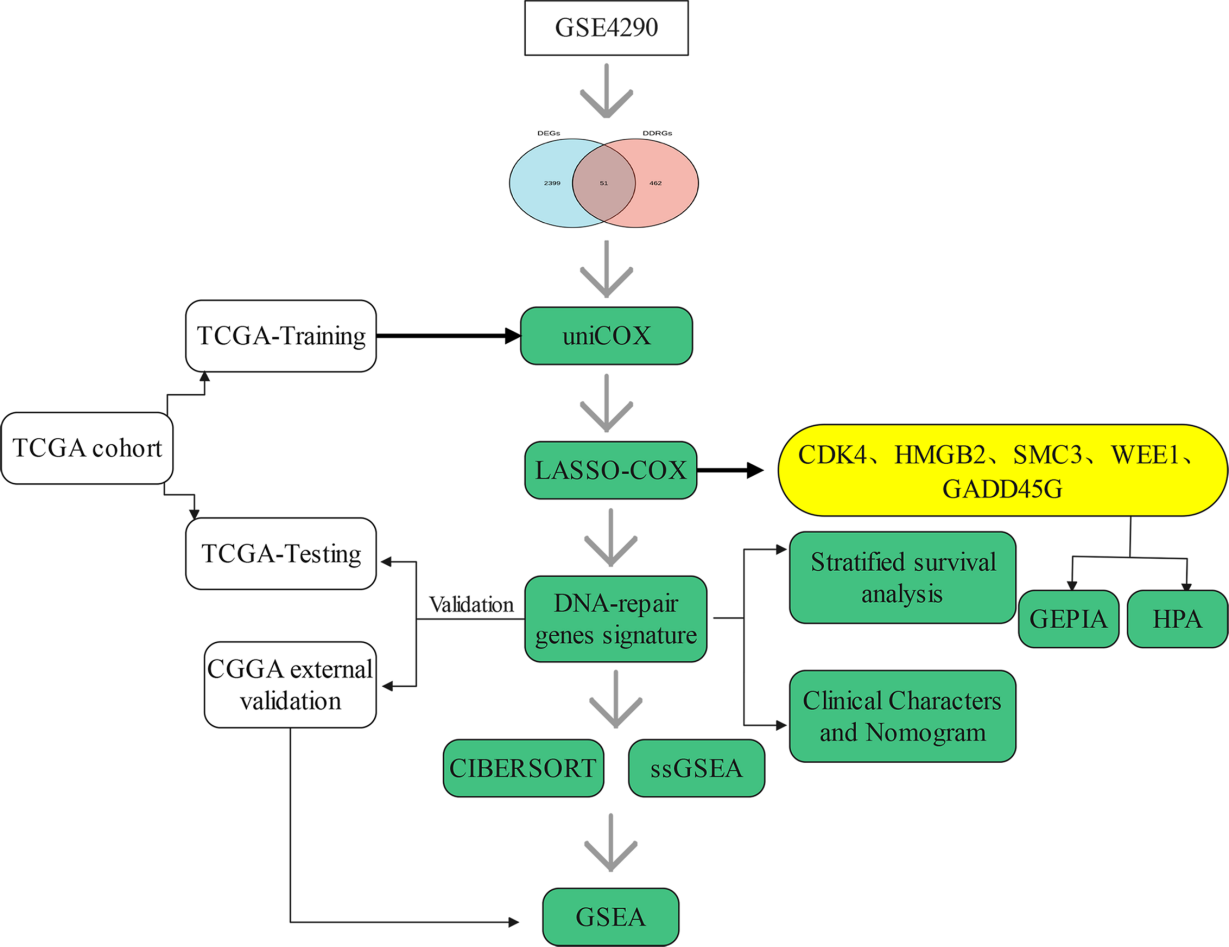
Flow chart of this study.

The flow chart of this research study is shown in **Figure 1**. Gene expression profile and clinical information of gliomas were obtained from clinical databases with large sample sizes. Patients with incomplete clinical information were removed. In total, 1,431 samples of glioma and 23 normal brain tissues were obtained from TCGA-LGG, TCGA-GBM, CGGA, and GSE4290 datasets. The detailed clinical features of gliomas collected from the articles are summarized in **Table 1**.

### Data Preprocessing and Identification of Candidate Genes

**Figure 2:**
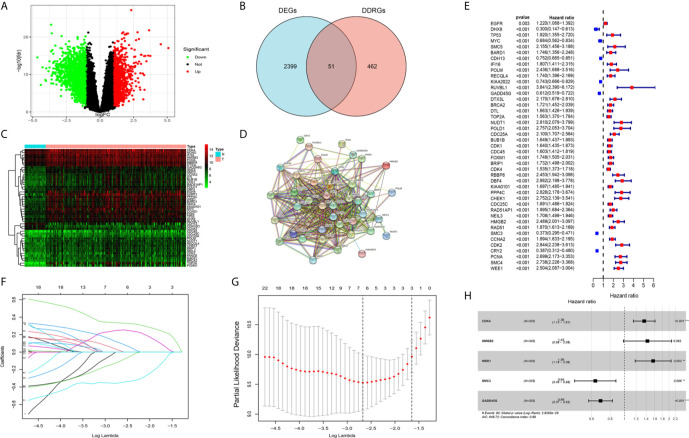
Screening candidate genes and construction a DNA damage repair genes (DDRGs) signature using LASSO regression and Cox proportional hazards regression. **(A)** Volcano plot of differentially expressed genes (DEGs) between gliomas and normal brain tissues. **(B)** Venn diagram showing the intersection of DEGs and DDRGs. **(C)** Heatmap was used to show the expression of the genes at the intersection. **(D)** The protein–protein interaction (PPI) network downloaded from the STRING database indicated the interactions among the candidate genes. **(E)** Forest plot showing the hazard ratios from the univariate Cox regression analysis. **(F)** LASSO coefficient profiles of the 43 candidate genes in TCGA training dataset. **(G)** A coefficient profile plot was generated against the log (lambda) sequence. Selection of the optimal parameter (lambda) in the LASSO model for TCGA training dataset. **(H)** Forest plot showing the five genes that composed the DDRGs signature. LASSO, least absolute shrinkage and selection operator; STRING, Search Tool for the Retrieval of Interacting Genes; TCGA, The Cancer Genome Atlas. **p < 0.01,***p < 0.001.

We analyzed differentially expressed genes (DEGs) between gliomas and normal brain tissues in the GSE4290 cohort using the Wilcoxon test; the microarray data included a total of 153 samples of glioma and 23 normal brain tissues. According to the screening criteria (|logFC|>1, false discovery rate <0.05), a total of 2,450 DEGs were identified. Among those, 1,450 were upregulated and 1,425 were downregulated (**Figure 2A**). In addition, 513 DDRGs were retrieved from the MSigDB (https://www.gsea-msigdb.org/gsea/msigdb) and previous literature (14) (**Supplementary Table S1**). We found that 51 genes were both DEGs and DDRGs (**Figure 2B**). The gene expression profiles of these genes are displayed through heatmaps (**Figure 2C**). **Figure 2D** illustrates the interaction of these 51 genes in the protein–protein interaction network. Next, TCGA data were randomly divided into two datasets (294 and 298 patients in the training and testing datasets, respectively) (**Table 1**). Univariate independent prognostic analysis was performed for these 51 genes in TCGA training dataset. A total of 43 genes were significantly associated with prognosis (**Figure 2E**). In addition, we used the Metascape website to analyze the function of these genes. As expected, these 43 DDRGs were involved in DNA damage repair processes and cell cycle pathways (**Supplementary Figure S1**).

### Construction of a Prognostic Model Using the Training Dataset

**Figure 3:**
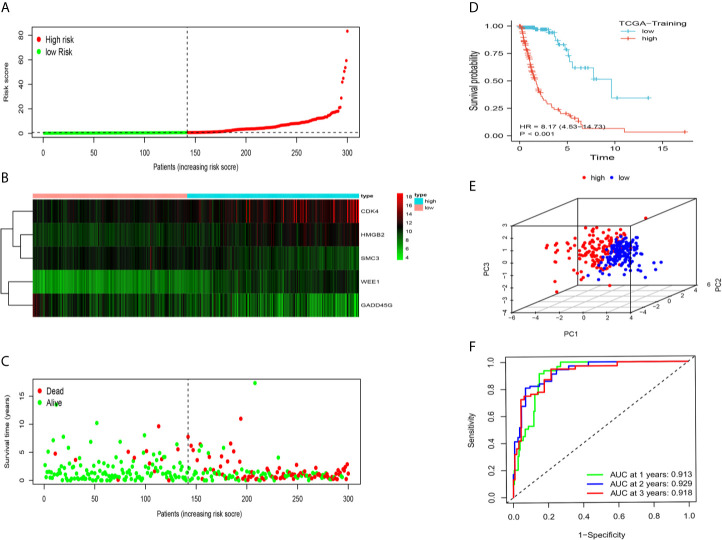
Prognostic analysis of the DDRG signature in TCGA training set. **(A)** The distribution and median value of the risk scores in TCGA training dataset. **(B)** The distributions of status, OS, and risk score in TCGA training dataset. **(C)** Heatmap showing the expression of five genes in the low- and high-risk groups. **(D)** Survival curve was used to analyze OS of the low- and high-risk groups in TCGA training dataset. **(E)** Principal components analysis (PCA) of the DDRGs signature. **(F)** The AUC values of time-dependent ROC curves verify the prognostic performance of the risk score in TCGA training dataset. AUC, area under the curve; DDRGs, DNA damage repair genes; OS, overall survival; ROC, receiver operating characteristic; TCGA, The Cancer Genome Atlas.

The 43 candidate genes were subsequently analyzed using LASSO regression and Cox proportional hazards regression. The independent variable in the regression was the expression profiles of these candidate genes, and the response variables were the clinical features of gliomas in TCGA training dataset (**Figures 2F, G**). Next, five risk genes (CDK4, HMGB2, WEE1, SMC3, and GADD45G) were identified (**Table 2**). The DDRGs signature was calculated as follows: RiskScore=0.307*CDK4_expression_+0.356*HMGB2_expression_+0.445*WEE1_expression_+(−0.453*SMC3_expression_)+(−0.371*GADD45G_expression_). The forest map of Cox regression analysis indicated that SMC3 and GADD45G were positively correlated with the prognosis of patients with glioma, whereas CDK4, WEE1, and HMGB2 were negatively correlated with prognosis (**Figure 2H**). To further investigate the properties of these five DDRGs, we retrieved their expression levels and impact on the prognosis of patients with glioma in the GEPIA website. In agreement with the results of a previous differential analysis, the expression levels of these five genes were higher in both LGG and GBM than in normal brain tissue (**Supplementary Figures S2A–E**). In terms of prognostic analysis, consistent with our previous results, high expression of CDK4, HMGB2, and WEE1 could lead to poor prognosis of glioma; however, gliomas with high expression of SMC3 and GADD45G were linked to longer survival (**Supplementary Figures S2F–J**). Subsequently, we investigated the proteins encoded by these five genes in patients with glioma using the Human Protein Atlas database. The previously described protein expression profile and gene expression levels were similar, and CDK4, HMGB2, WEE1, SMC3, and GADD45G exhibited medium staining intensity. However, their corresponding expression levels in normal brain tissues were not detected, or the staining intensity was low (**Supplementary Figures S3A–E**). Each patient could be scored according to this formula; patients with glioma in TCGA training dataset were divided into high-risk and low-risk groups (n=147, respectively) according to the median score (**Figure 3A**). A heatmap was used to describe the expression of these five DDRGs in different groups (**Figure 3B**). In the high-risk group, CDK4, HMGB2, and WEE1 were upregulated, whereas SMC3 and GADD45G were downregulated. **Figure 3C** suggests that patients in the high-risk group were associated with a higher mortality rate and shorter survival than those in the low-risk group. Consistently, based on the Kaplan–Meier curve, patients in the low-risk group had a significantly better OS (hazard ratio [HR]=8.17, 95% confidence interval [CI]: 4.53–14.73, P<0.001) than those in the high-risk group (P<0.01) (**Figure 3D**). Following the classification of patients with glioma into high- and low-risk groups according to the risk model, PCA was performed. The results revealed that the patients in different risk groups were distributed in two directions (**Figure 3E**). Time-dependent ROC curves were constructed to further evaluate the accuracy of the DDRGs signature for predicting prognosis. The area under the curve values for 1-, 2-, and 3-year survival were 0.913, 0.929, and 0.918, respectively (**Figure 3F**). The calculated C index was to be 0.769 in the training TCGA cohort.

**Table 2:** The information of 5 DNA repair genes constructing the prognostic risk model.

Gene symbol	Description	Ensemble ID	Category	Coeffcient	HR (95%CI)	Pvalue
CDK4	Cyclin Dependent Kinase 4	ENSG00000135446	Protein Coding	0.31	1.36 (1.15-1.61)	<0.01
HMGB2	High Mobility Group Box 2	ENSG00000164104	Protein Coding	0.36	1.43 (0.98-2.08)	0.06
WEE1	WEE1 G2 Checkpoint Kinase	ENSG00000166483	Protein Coding	0.45	1.56 (1.18-2.06)	<0.01
SMC3	Structural Maintenance Of Chromosomes 3	ENSG00000108055	Protein Coding	-0.45	0.64 (0.46-0.88)	0.01
GADD45G	Growth Arrest And DNA Damage Inducible Gamma	ENSG00000130222	Protein Coding	-0.37	0.69 (0.57-0.83)	<0.01

### Analysis of the DDRGs Signature Using TCGA Testing and CGGA Validation Datasets

**Figure 4:**
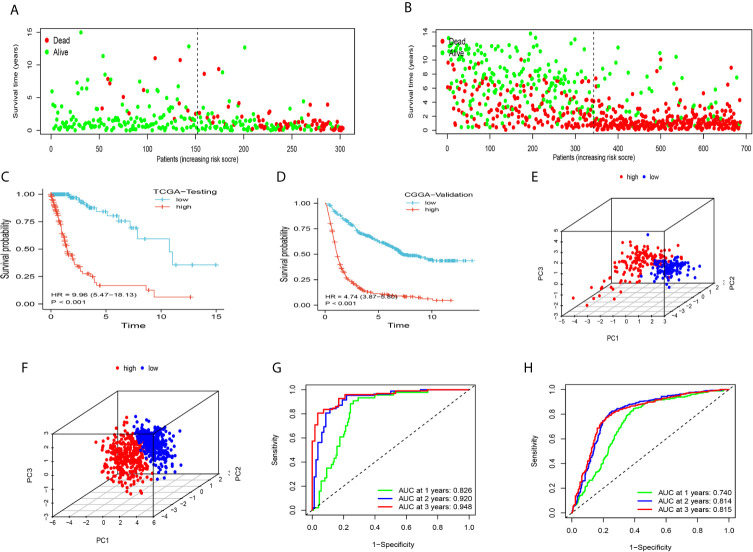
The accuracy of the DDRGs signature was verified using internal and external validation. **(A, B)** Scatter plots depicting the survival and status of patients in the high- and low-risk groups in TCGA testing and CGGA validation datasets. **(C, D)** Survival curve was used to analyze OS in the low- and high-risk groups in TCGA testing and CGGA sets. **(E, F)** Principal component analysis (PCA) of the DDRG signature in TCGA testing and CGGA sets. **(G, H)** ROC curves were constructed for TCGA testing and CGGA sets. CGGA, Chinese Glioma Genome Atlas; DDRG, DNA damage repair gene; OS, overall survival; ROC, receiver operating characteristic; TCGA, The Cancer Genome Atlas.

We further clarified the role of the DDRGs signature in predicting prognosis in patients with glioma by performing analysis using TCGA testing and CGGA validation datasets. Scores for each patient were calculated using the formula shown above, and patients were divided into high- and low-expression groups based on the corresponding median score (**Supplementary Figures S4A–D**). The distribution of patients in TCGA testing and CGGA cohorts was presented in **Figures 4A, B**. High-risk patients in the TCGA training dataset had a shorter OS (HR=9.96, 95% CI: 5.47–18.13, P<0.001), in the external CGGA validation dataset were same as TCGA dataset (HR=4.74, 95% CI: 3.87–5.80, P<0.001) (**Figures 4C, D**). PCA revealed that patients in the two subgroups were distributed in discrete directions in both TCGA testing and CGGA datasets (**Figures 4E, F**). In addition, in the TCGA testing dataset, the area under the curve values of the DDRGs signature for 1, 2, and 3 years were 0.826, 0.920, and 0.948, respectively; in the CGGA dataset, these values were 0.740, 0.814, and 0.815, respectively (**Figures 4G, H**). The C-indexes were 0.722 and 0.819 in testing TCGA and validation CGGA sets.

### Independent Prognostic Value of the DDRGs Signature

Univariate and multivariate Cox regression analyses were performed to assess whether DDRGs signature was an independent prognostic indicator. In the TCGA database, Univariate Cox regression analysis demonstrated that the risk scores were associated with the overall survival rate of glioma patients(HR=5.103, 95%CI=3.407-7.620, P<0.001). Multivariate Cox regression analysis revealed that the risk scores were independent risk factors for predicting the overall survival rate of glioma patients(HR=2.967, 95%CI=1.627-4.823, P<0.001). The results were validated in the CGGA (**Supplementary Figures S5A–D**).

### Prediction of Outcome by the DDRGs Signature in Stratified Patients

**Figure 5:**
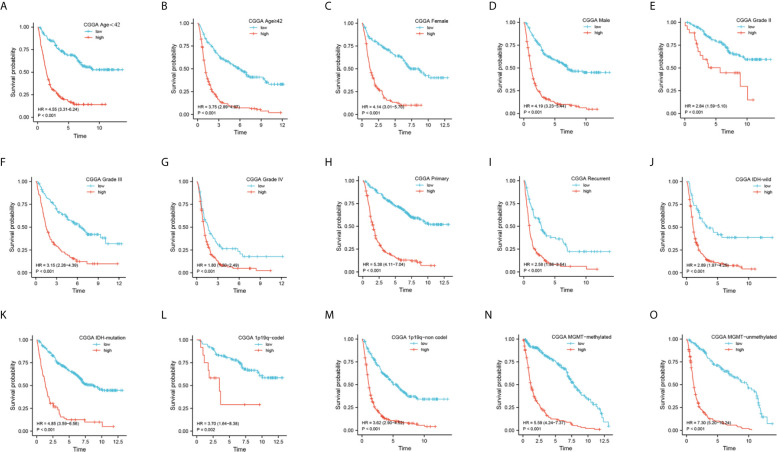
Prediction of outcome of the DDRGs signature in stratified patients in the CGGA dataset. **(A–O)** Survival analysis of the signature in patients stratified by age, sex, PRS type, grade, IDH, 1p19q status, and MGMT promoter. CGGA, Chinese Glioma Genome Atlas; DDRG, DNA damage repair gene; IDH, isocitrate dehydrogenase; MGMT, O-6-methylguanine-DNA methyltransferase; PRS, primary-recurrent-secondary.

We subsequently sought to validate the prognostic role of the DDRGs signature in gliomas with different clinical characteristics. Survival analysis was performed for patients from the CGGA dataset who were divided according to their age (<42, ≥42), sex (female, male), PRS type (primary, recurrent), grade (II, III, IV), IDH (mutation, wild), 1p19q (co-deletion [codel], non-codel), and MGMT status (methylated, unmethylated). The Kaplan–Meier analysis showed that a low risk score was linked to longer OS than a high risk score in all stratified patients (**Figures 5A–O**). Consistent results were obtained from TCGA dataset (**Supplementary Figures S6A–J**). Interestingly, we noted that all stratified patients with grade IV disease in TCGA dataset were in the high-risk subgroup. Moreover, the risk scores were significantly higher in GBM than LGG in the GSE4290, TCGA, and CGGA cohorts (**Supplementary Figures S7A–C**). These results suggested that the DDRGs signature could precisely predict the prognosis of gliomas, linking patients with a high-risk score to poor survival.

### Relationship Between the DDRGs Signature and Clinical Characteristics of Gliomas

**Figure 6:**
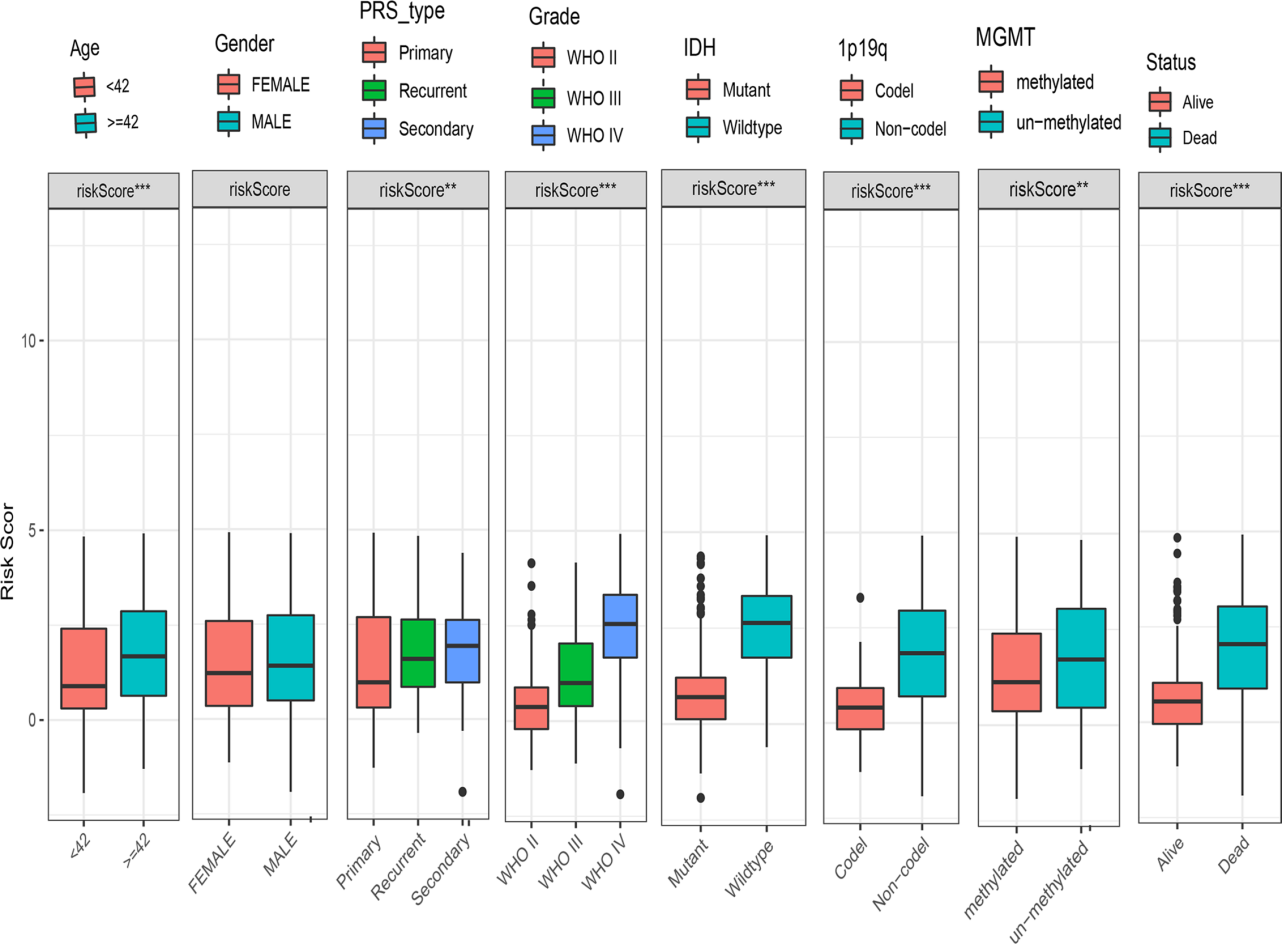
Box charts showing the characteristics of patients in different risk groups, including age, sex, PRS type, grade, IDH, 1p19q, MGMT, and survival status. IDH, isocitrate dehydrogenase; MGMT, O-6-methylguanine-DNA methyltransferase; PRS, primary-recurrent-secondary.

To examine the association between this DDRGs signature and clinical characteristics of gliomas in CGGA cohort, we investigated potential positive correlations of the risk score with age and grade of glioma. We also explored that the risk score was lower in IDH mutation, 1p19q codel and MGMT methylated significantly which were the clinical features with good prognosis of glioma (**Figure 6**).

### Relationship Between the DDRGs Signature and Immune Cell Infiltration in the Tumor Microenvironment

**Figure 7:**
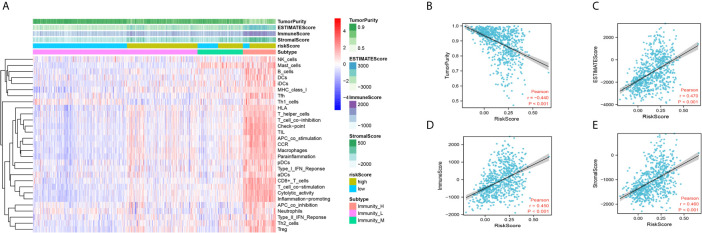
Immune infiltration patterns of low- and high-risk score analyzed by ssGSEA methods in glioma from the CGGA dataset. **(A)** Heatmap revealing the scores of immune cells in low, middle, and high immunities. **(B–E)** Scatter plot showing the correlation between risk score and tumor purity, ESTIMATE, immune and stromal score. CGGA, Chinese Glioma Genome Atlas; ssGSEA, single-sample gene set enrichment analysis.

**Figure 8:**
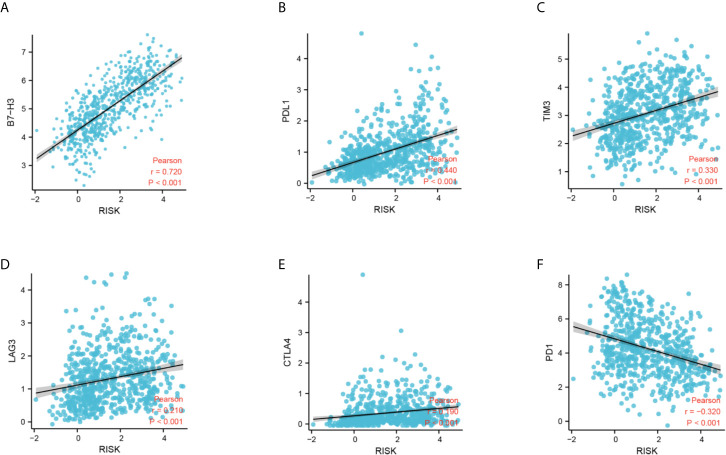
Correlation analysis between the DDRG signature and immune checkpoints. **(A–F)** Relationship between the DDRG signature and B7-H3, PDL1, TIM3, LAG3, CTLA4, and PD1, respectively. CTLA4, cytotoxic T-lymphocyte associated protein 4; DDRG, DNA damage repair gene; LAG3, lymphocyte activating 3; PD1, programmed cell death 1; PDL1, programmed cell death 1 ligand 1; TIM3, T-cell immunoglobulin mucin family member 3.

Next, we performed CIBERSORT and ssGSEA to evaluate the relationship between the DDRGs signature and immune cell infiltration in the tumor microenvironment of gliomas. The 686 glioma samples obtained from the CGGA dataset were segregated into low- and high-risk group using the formula shown above. CIBERSORT was used to analyze the RNA sequencing data of these selected patients and evaluate the fractions of 22 immune cell types in the high- and low-risk groups. Surprisingly, the risk score was positively correlated with T follicular helper cells, regulatory T (Treg) cells, and macrophages M0 (P<0.01). In contrast, it was negatively correlated with CD4 naïve T cells, CD4 memory resting T cells, gamma-delta T cells, monocytes, and activated mast cells (P<0.01) (**Supplementary Figures S8A, B**). Subsequently, we also conducted ssGSEA to determine the degree of immune cell infiltration. We similarly observed that the risk score was positively correlated with activated dendritic cells, B cells, CD8+ T cells, dendritic cells, macrophages, mast cells, plasmacytoid dendritic cells, T helper (Th) cells, Th2 cells, tumor-infiltrating lymphocytes, and Treg cells; in contrast, the risk score was negatively correlated with neutrophils and Th1 cells. Moreover, we found that the risk score was correlated with immune processes, such as CCR, check-point, cytolytic activity, etc. (**Supplementary Figures S8C, D**). According to these results, we divided patients with glioma into high- group, medium-, and low-immunity groups. As expected, patients with higher risk scores tended to be in the high-immunity group (**Supplementary Figure S8E**). In addition, the present study revealed that the risk score was negatively correlated with tumor purity, and positively correlated with immune score and stroma score. These findings indicated that the risk score was closely related to the tumor microenvironment (**Figures 7A–E**). Next, we analyzed the relationship between the risk score and immune checkpoints (programmed cell death 1 [PD1], programmed cell death 1 ligand 1 [PDL1], T-cell immunoglobulin mucin family member 3 [TIM3], cytotoxic T-lymphocyte associated protein 4 [CTLA4], B7-H3, and lymphocyte activating 3 [LAG3]) through correlation analysis. The Pearson analysis showed that the risk score was strongly positively correlated with B7-H3 (r=0.720), moderately and weakly correlated with PDL1 (r=0.443), TIM3 (r=0.330), LAG3 (r=0.210), and CTLA4 (r=0.190), and moderately negatively correlated with PD1 (r=−0.320) (**Figures 8A–F**).

### Identification of Involved Signaling Pathways

**Figure 9:**
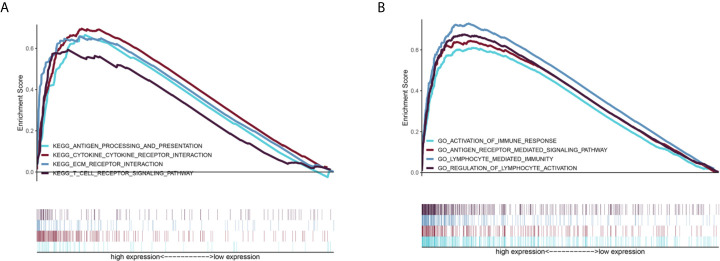
GSEA of the relevant mechanisms involved in the DDRG signature. KEGG **(A)** and GO **(B)**. DDRGs, DNA damage repair genes; GSEA, gene set enrichment analysis; KEGG, Kyoto Encyclopedia of Genes and Genomes; GO, Gene Ontology.

GSEA was performed to further examine the mechanism of DDRGs and poor prognosis of patients with glioma. In the c2.cp.kegg dataset, we found that the high-risk group was significantly associated with the following pathways: antigen processing and presentation (NES=2.80, P<0.0001), cytokine-cytokine receptor interaction (NES=1.937, P=0.0039), extracellular matrix receptor interaction (NES=1.90, P=0.0059), and T cell receptor signaling pathway (NES=1.703, P=0.017). Gene Ontology analysis showed that high risk was closely associated with the following pathways: activation of immune response (NES=2.79, P<0.0001), antigen receptor-mediated signaling pathway (NES=1.96, P<0.0012), lymphocyte-mediated immunity (NES=2.13, P<0.0001), and regulation of lymphocyte activation (NES=2.330, P<0.0001) (**Figures 9A, B**).

### A Personalized Prognostic Prediction Model

**Figure 10:**
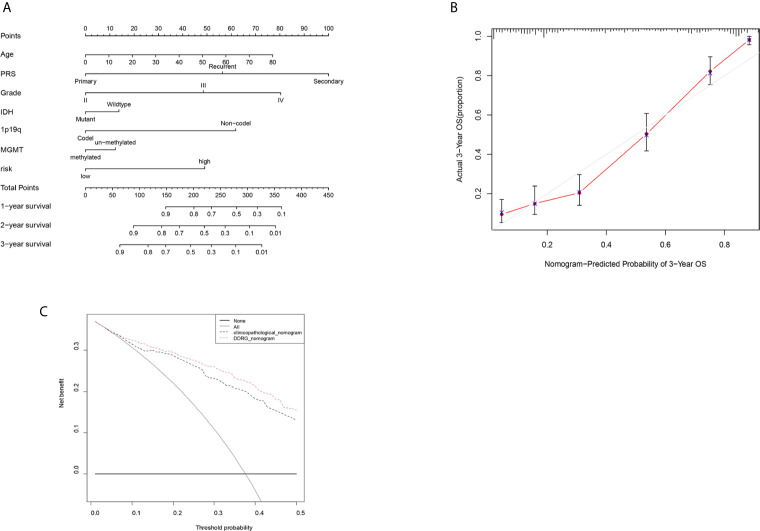
Nomogram for the prediction of prognostic probabilities in the CGGA dataset. **(A)** The nomogram for the prediction of OS was developed using the CGGA dataset. **(B)** The calibration plots for predicting 3-year survival. **(C)** Decision curve analysis for the DDRGs signature nomogram and the clinicopathological nomogram to estimate the OS. CGGA, Chinese Glioma Genome Atlas; OS, overall survival; DDRGs, DNA damage repair genes.

The nomogram can conveniently and rapidly predict the prognosis of patients with cancer; hence, it is widely used in clinical research on cancer (19). According to the clinical information and risk model score of patients with glioma, a nomogram model was established to predict the prognosis of patients, including age, PRS type, grade, IDH, 1p19q, MGMT status, and risk score. Each patient was scored according to their respective different clinical traits and risk score; subsequently, the 1-, 2-, and 3-year survival rates of patients with glioma were predicted according to the prediction line at the bottom of the nomogram (**Figure 10A**). Calibration curves indicated that actual and predicted survival matched well (**Figure 10B**), particularly for 3-year survival. Meanwhile, the calculated C index was to be 0.785. The decision curves shown in **Figure 10C** demonstrated the clinical usefulness of the prediction models, indicating that the DDRGs nomogram achieved a higher overall net benefit than the clinicopathologic nomogram within most range of threshold probabilities.

## Discussion

In 2020, the number of new CNS tumor cases was 308,102, accounting for 1.6% of the total number of new tumor cases, worldwide. Moreover, 251,329 deaths due to brain tumors were reported, accounting for 2.5% of cancer-related deaths globally (22). Gliomas constitute approximately 70% of intracranial malignancy cases and are the leading cause of death among these patients. The prognosis of patients with gliomas, particularly GBM, is poor; median survival after the postoperative administration of concurrent chemoradiotherapy is <2 years (23). The main reasons for the poor prognosis of gliomas are the aggressive proliferation, invasive growth and chemoradiotherapy resistance of glioma cells (24).

It has been proved that DDRGs are closely related to chemoradiotherapy resistance of tumors (25, 26). In this study, we investigated DDRGs and constructed a risk model consisting of five such genes (CDK4, HMGB2, WEE1, SMC3, and GADD45G) by differential analysis using LASSO regression and Cox proportional hazards regression. We validated the accuracy of this model for predicting the prognosis of gliomas by internal and external validation and stratification analysis. In the TCGA cohort, we found that all GBM patients were in high-risk subgroup. But we did not observe it in the CGGA set. Nevertheless, we found that most of the patients with GBM in the CGGA set were still in high-risk subgroup and had statistical significance. Further, we also explored that the risk score of LGG was significantly lower than GBM in TCGA, CGGA and GSE4290 databases. Moreover, by analyzing the clinical characteristics of patients with glioma, we found that the risk score was positively correlated with malignancy and negatively correlated with protective factors (e.g., IDH mutation, 1p19q codel, and MGMT methylation). Collectively, these results suggest that this risk model is significantly associated with the prognosis of patients with glioma.

The CDK4 promotes tumor cell differentiation from G1 to S phase and increases proliferation. Schmidt et al. (27) were the first to report that amplification of the CDK4 gene occurs in highly malignant GBMs and anaplastic astrocytomas, whereas there are alterations observed in benign astrocytomas. Additionally, recent studies have shown that overexpression of the CDK4 gene was closely related to poor prognosis of patients with glioma (28). HMGB2 is a member of the high mobility group of the nonhistone chromatin-associated proteins that regulate the processes of transcription, replication, recombination, and DNA repair (29). HMGB2 is highly expressed during embryonic development, but lowly expressed in adult organs; it is mainly detected in lymphoid organs and testes. HMGB2 is highly expressed in most tumor tissues (30). As a target gene of mi-R130a, HMGB2 controls the proliferation and epithelial mesenchymal transition of glioma cells (31). WEE1 is a nuclear kinase belonging to the serine/threonine protein kinase family. It is a key regulator of cell cycle progression and maintenance of genomic stability. WEE1 can control the cell cycle by phosphorylating CDK1 and regulating the activity of the CDK1/cyclin B complex. As an integral part of the G2/M phase checkpoint, WEE1 determines the time point of entry into mitosis and inhibits the early progression of the cell cycle, while also participating in the cellular response to DNA damage (32). Shahryar et al. (33) reported that inhibition of WEE1 expression increased the sensitivity of GBM to radiation. This effect may be related to the blockage of WEE1 in the G2M phase of GBM. SMC3 is an essential component of the cohesin family. Studies found that SMC3 activates nuclear factor-κB (NF-κB) through autocrine tumor necrosis factor-α (TNF-α), which inhibits apoptosis in cancer cells. However, it also can activate the AKT pathway, thereby promoting cancer growth (34). GADD45G (relative molecular mass: 18,000 Da) is an evolutionarily conserved protein among members of the growth arrest and DNA damage-inducible protein 45 family. Its expression is elevated in response to a variety of exogenous genotoxic and oncogenic stimuli. Li et al. (35) found that knockdown of GADD45G expression increased the proliferative and migratory ability of esophageal cancer cells. It has also been shown that overexpression of GADD45G reverses resistance to sorafenib both *in vitro* and *in vivo*. Moreover, the proapoptotic effect of sorafenib on sorafenib-sensitive cells is partially abolished by inhibiting the expression of GADD45G using siRNA (36). The traditional view that the CNS is an immune privileged organ arose from the fact that the blood–brain barrier can selectively block the delivery of immune cells and immune macromolecules from the periphery to the CNS. Nonetheless, this is not absolute, and recent studies suggested that lymphatic vessels existing in the brain can also generate an immune response (37). The use of immunotherapy has resulted in significant breakthroughs in the therapeutic landscape of cancer, particularly in the treatment of tumors, such as lung cancer, melanoma, and colorectal cancer. Nevertheless, in gliomas, immunotherapy has not achieved good outcomes, mainly because glioma gradually develops a multifactorial, multicellular complex suppressive immune microenvironment during growth (9, 38). Infiltration of immunosuppressive cells in the tumor microenvironment is key to the efficacy of immunotherapy and tumor immune evasion; the major immunosuppressive cells include tumor-associated macrophages (TAMs) and Treg cells (39). In this study, the infiltration of immune cells in each patient was determined through CIBERSORT and ssGSEA; the high-risk group had more immunosuppressive cells, such as TAMs and Treg cells. Patients were divided into high-, moderate-, and low-immunity groups, according to the degree of immune cell infiltration. Unexpectedly, we observed a trend of positive association between patients in the high-risk group and high-immunity group. It has also been shown that patients with glioma and high immunity are associated with worse prognosis (40). The present findings are consistent with those of previous studies. This also explains the poorer prognosis of patients in the high-risk group.

Chemoradiotherapy can cause double-strand breaks in the DNA of cancer cells, thereby initiating a series of reexamined biochemical reactions for the repair of damaged DNA. Failure to efficiently repair damage or correctly modify DNA can lead to gene mutations, chromosomal rearrangements, or even cell death. Therefore, DNA damage repair is intimately linked to genomic instability. Genomic instability in cells can result in the production of a large number of neoantigens that are more easily recognized by the immune system, which in turn leads to the infiltration of more immune cells in the tumor microenvironment. The infiltrating immune cells are reprogramed by various tumor-derived cytokines and chemokines, acquiring unique functional phenotypes and transforming into tumor-related immune cells. The tumor microenvironment of glioma is particular owing to the unique brain immunology. As the essence of blood–brain barrier, the immune privilege of CNS can inhibit and delay the immune response. The occurrence, development, and immune evasion of gliomas are closely related to this physiological state (i.e., the tumor immunosuppressive microenvironment), which markedly limits the effectiveness of immunotherapy. In addition to the inherent immunosuppressive microenvironment of glioma, the infiltration of immunosuppressive cells (e.g., TAMs, myeloid-derived suppressor cells, tumor-associated neutrophils, and Treg cells) is also closely related to the tumor immunosuppressive microenvironment (41). Glioma cells interact with various components in their microenvironment, jointly inducing the formation of the immunosuppressive microenvironment and promoting the progression of glioma. Therefore, elucidation of the mechanism of the immunosuppressive microenvironment of glioma may provide an important theoretical basis for improving immunotherapy strategies against glioma. Of the various immunotherapies, checkpoint blockade is currently the most widely applied in clinical practice; however, it may not be the most promising treatment for glioma. Effector T cells can be reactivated by binding to specific antibodies and checkpoint molecules, thereby performing their cytotoxic role against tumor cells.

PD1 and its ligand PDL1/2 are the most widely studied immune checkpoint molecules thus far. These molecules can negatively regulate the signal transduction pathway mediated by T cell receptors. By binding to PDL1, PD1 suppresses the proliferation and differentiation of T cell, blocks the production of inflammatory factors, and leads to T cell inactivation. CTLA4, a member of the immunoglobulin superfamily, is a glycoprotein expressed on the surface of activated clusters of differentiated CD4 and CD8 T cells. It is an important negative regulator in the immune system and the first immunomodulatory molecule to be used in targeted therapy. CTLA4 inhibits T cell activation and induces T cell incompetence by binding the natural CD80 and CD86 ligands expressed on antigen-presenting cells. LAG3 is a member of the immunoglobulin superfamily and exerts an inhibitory effect on lymphocytes. It can enhance the negative regulatory function of Treg cells, which play an important role in the immune response. In addition, it is involved in the immune evasion of various tumor cells. TIM3 is a negative immune checkpoint molecule that can be expressed in T cells, monocyte macrophages, Treg cells, natural killer cells, and tumor cells. The binding of TIM3 to its ligand galectin 9 (LGALS9) induces the depletion of T cells, which cannot be activated and are unable to secrete cytokines, leading to tumor immunosuppression and immune evasion (42). As a newly discovered member of the B7-CD28 checkpoint pathway, B7-H3 plays an extremely important role in the process of tumor immunity. Numerous studies have shown that B7-H3 is highly expressed in most tumors and associated with tumor immune evasion, which is inextricably linked to tumor stemness, invasion, and metastasis (43). B7-H3 is highly expressed in patients with glioma, positively correlated with tumor pathological grade, and negatively correlated with survival (44). In this study, we found that the DDRGs signature was positively correlated with these immune checkpoints, particularly B7-H3, which could serve as a potential approach to enhancing immunotherapy for glioma.

The present study was characterized by some limitations. Firstly, despite the use of the GEO, TCGA, and CGGA databases for validation, there is a lack of multicenter data. In future studies, we plan to analyze data of patients with glioma from our hospital and other hospitals. Secondly, our results warrant further validation *in vitro* and *in vivo*. Further validation of this risk model could lead to precise prediction of the prognosis of patients with glioma and provide guidance for treatment.

In this study, we analyzed the differential expression of DDRGs between gliomas and normal brain tissues and constructed a signature composed of five such genes. The DDRGs signature could accurately predict the prognosis of patients with glioma, which was validated using data obtained from both TCGA and CGGA databases. Most importantly, immune cell infiltration analysis revealed that the DDRGs signature was closely related to inhibitory immune cell infiltration in the microenvironment of glioma. These findings provide a new potential approach and strategy for improving immunotherapy against glioma.

## Data Availability Statement

The datasets presented in this study can be found in online repositories. The names of the repository/repositories and accession number(s) can be found in the article/**Supplementary Material**.

## Ethics Statement

Ethical review and approval was not required for the study on human participants in accordance with the local legislation and institutional requirements. Written informed consent for participation was not required for this study in accordance with the national legislation and the institutional requirements.

## Author Contributions

XX was responsible for the overall design of this study. GW, HZ, and LT analyzed the data and edited the manuscript. TY revised the images and tables of this article. XH and PC performed R language modification. HL validated the data analysis. WW and ZX revised the discussion of the article. LH contributed to the study design. All authors contributed to the article and approved the submitted version.

## Funding

This study was supported by the Key R&D program of Hebei Province (Grant number: 19277737D).

## Conflict of Interest

The authors declare that the research was conducted in the absence of any commercial or financial relationships that could be construed as a potential conflict of interest.

## Supplementary Material

Supplementary Figure 1 Metascape dataset showing the interaction and enrichment pathways of candidate genes.

Supplementary Figure 2 The gene expression of the constructed risk model and its prognostic impact on glioma patients were analyzed through the GEPIA online website. Figure 2 A-E exhibited the differential expression of CDK4, HGMB2, WEE1, SMC3, GADD45G respectively in LGG and GBM. Figure 2 F-J revealed the overall survival of low and high expression of CDK4, HGMB2, WEE1, SMC3, GADD45G in glioma, respectively. GEPIA, Gene Expression Profiling Interactive Analysis; LGG, lower grade glioma; GBM, glioblastoma.

Supplementary Figure 3 Protein expression of the genes used for the construction of risk models by the Human Protein Atlas. A-E: immunohistochemical assay in normal and tumor tissue of CDK4, HGMB2, WEE1, SMC3, GADD45G, respectively.

Supplementary Figure 4 TCGA testing and CGGA datasets were used to verify the accuracy of the DDRGs signature. **(A, B)** Scatterplots illustrating the risk scores for patients with glioma in TCGA testing and CGGA datasets. **(C, D)** The Heatmap illustrating the expression of the five genes in the high- and low-risk groups. TCGA, The Cancer Genome Atlas; CGGA, Chinese Glioma Genome Atlas; DDRGs, DNA damage repair genes.

Supplementary Figure 5 Univariate and multivariate Cox analysis of prognostic risk scores for gliomas. Univariate Cox regression analysis. Forest plot of associations between risk factors and the survival of gliomas in the TCGA **(A)**, and CGGA **(B)**. Multiple Cox regression analysis. The DDRGs signature is an independent predictor of gliomas in the TCGA** (C)**, and CGGA **(D)**. TCGA, The Cancer Genome Atlas; CGGA, Chinese Glioma Genome Atlas.

Supplementary Figure 6 Prediction of outcome of the DDRGs signature in stratified patients in TCGA dataset. Survival analysis of the signature in patients stratified by age **(A, B)**, gender **(C, D)**, grade **(E, F)**, IDH **(G, H)**, and 1p19q status **(I, J)**. DDRGs, DNA damage repair genes; TCGA, The Cancer Genome Atlas; IDH, isocitrate dehydrogenase.

Supplementary Figure 7 Comparison of risk scores in GBM and LGG in the TCGA **(A)**, CGGA **(B)**, and GSE4290 **(C)**. TCGA, The Cancer Genome Atlas; CGGA, Chinese Glioma Genome Atlas.

Supplementary Figure 8 Relationship between the DDRGs signature and immune infiltrating cells in the tumor microenvironment. **(A)** CIBERSORT was used to analyze the types and proportion of infiltrating immune cells in the tumor microenvironment of patients with glioma. **(B)** Differences in immune infiltrating cells between high- and low-risk groups determined by CIBERSORT. **(C, D)** Differences in immune infiltrating cells and immune related pathways between high- and low-risk groups determined by ssGSEA. **(E)** Violin diagram showing the relationship between immune grouping and risk score. DDRGs, DNA damage repair genes; ssGSEA, single-sample gene set enrichment analysis.
