# Supplementary figures and images for: A Prognostic DNA Damage Repair Genes Signature and Its Impact on Immune Cell Infiltration in Glioma
Source: Front Oncol. 2021 May 28;11:682932. doi: 10.3389/fonc.2021.682932 (PMC8193723; doi:10.3389/fonc.2021.682932)

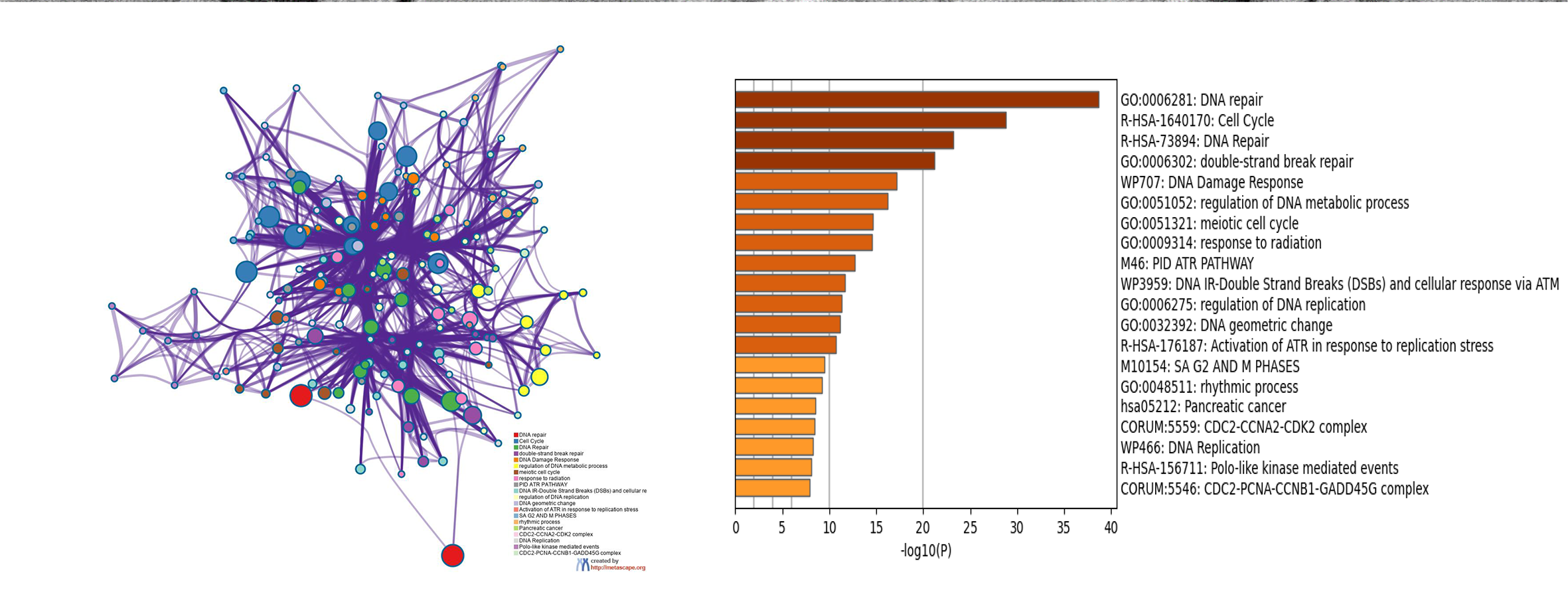

Supplement: Supplementary Figure 1 — Metascape dataset showing the interaction and enrichment pathways of candidate genes. [file Image_1.tif]

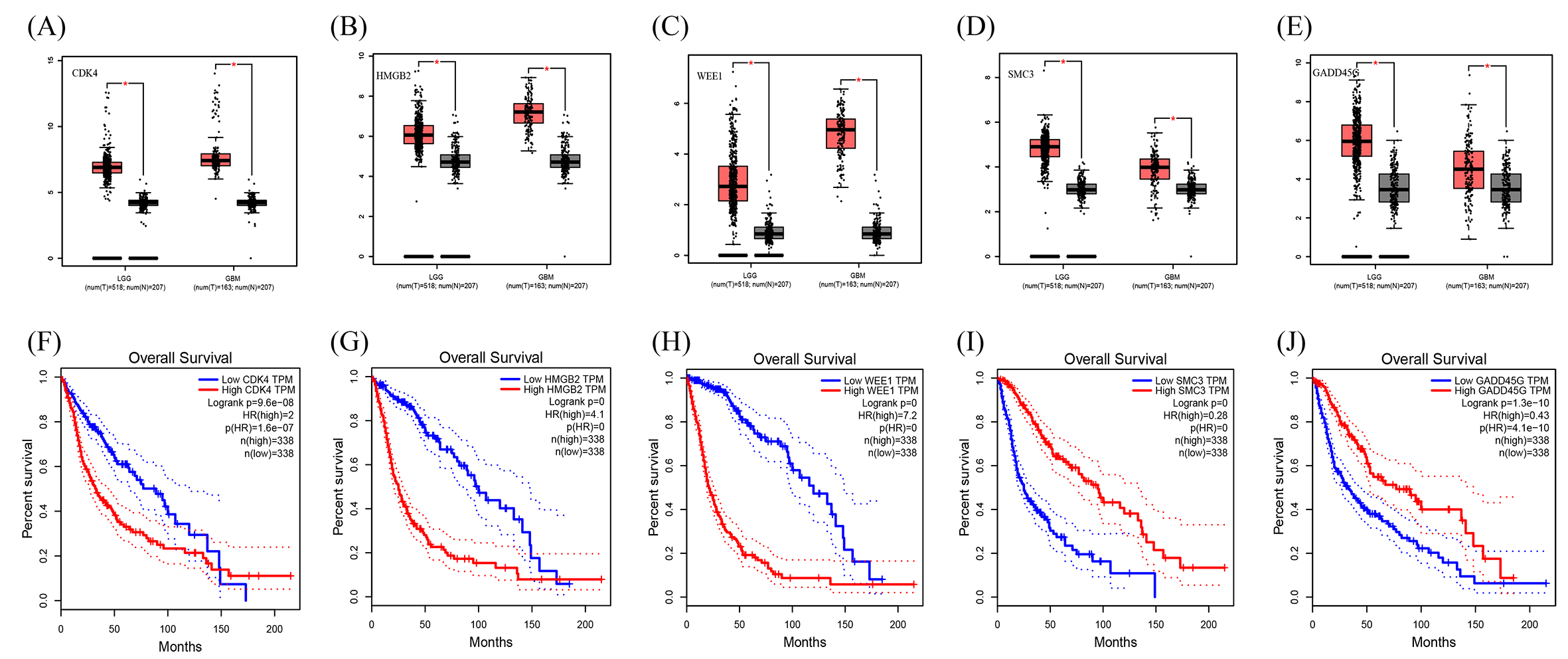

Supplement: Supplementary Figure 2 — The gene expression of the constructed risk model and its prognostic impact on glioma patients were analyzed through the GEPIA online website. Figure 2 A-E exhibited the differential expression of CDK4, HGMB2, WEE1, SMC3, GADD45G respectively in LGG and GBM. Figure 2 F-J revealed the overall survival of low and high expression of CDK4, HGMB2, WEE1, SMC3, GADD45G in glioma, respectively. GEPIA, Gene Expression Profiling Interactive Analysis; LGG, lower grade glioma; GBM, glioblastoma. [file Image_2.tif]

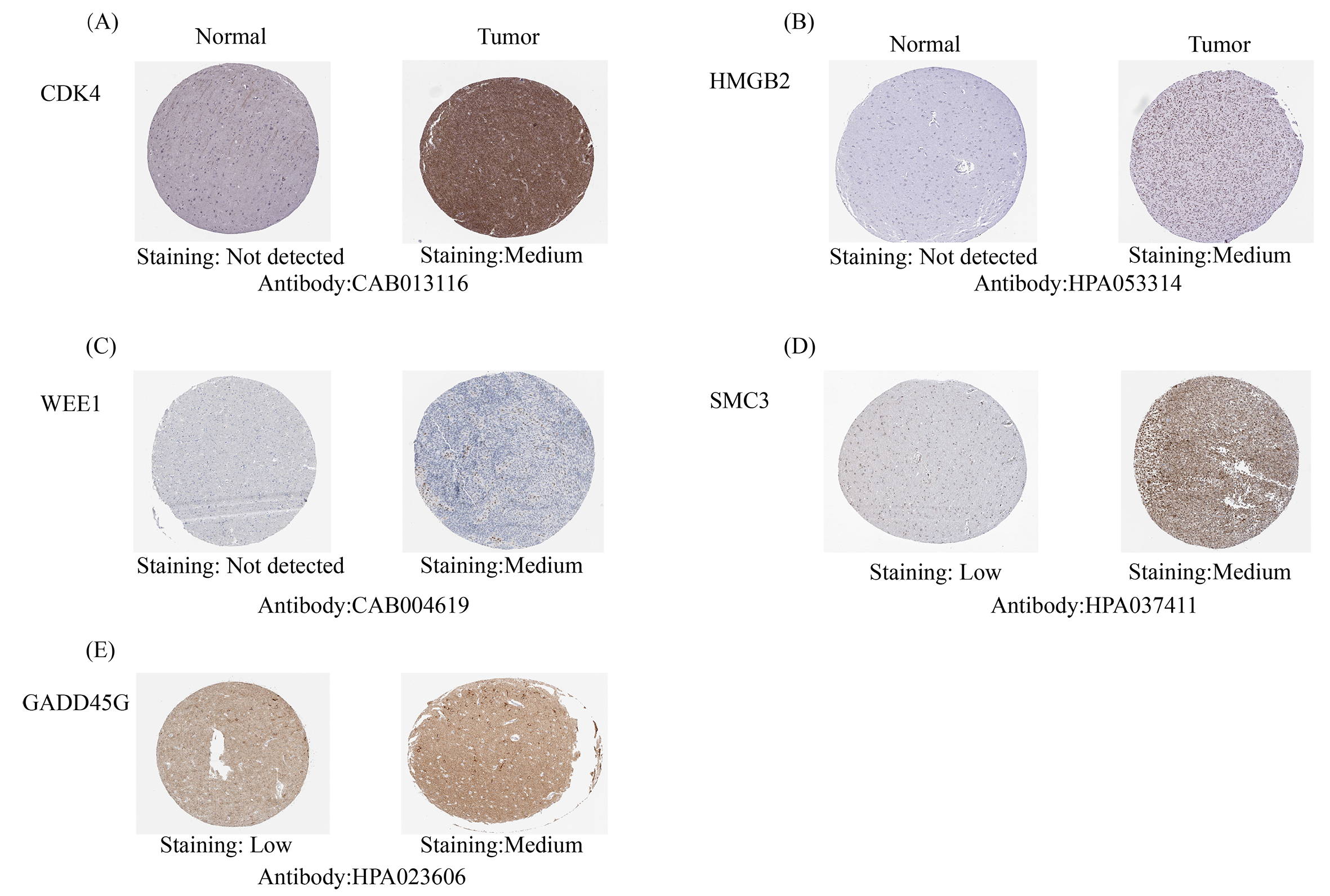

Supplement: Supplementary Figure 3 — Protein expression of the genes used for the construction of risk models by the Human Protein Atlas. A-E: immunohistochemical assay in normal and tumor tissue of CDK4, HGMB2, WEE1, SMC3, GADD45G, respectively. [file Image_3.tif]

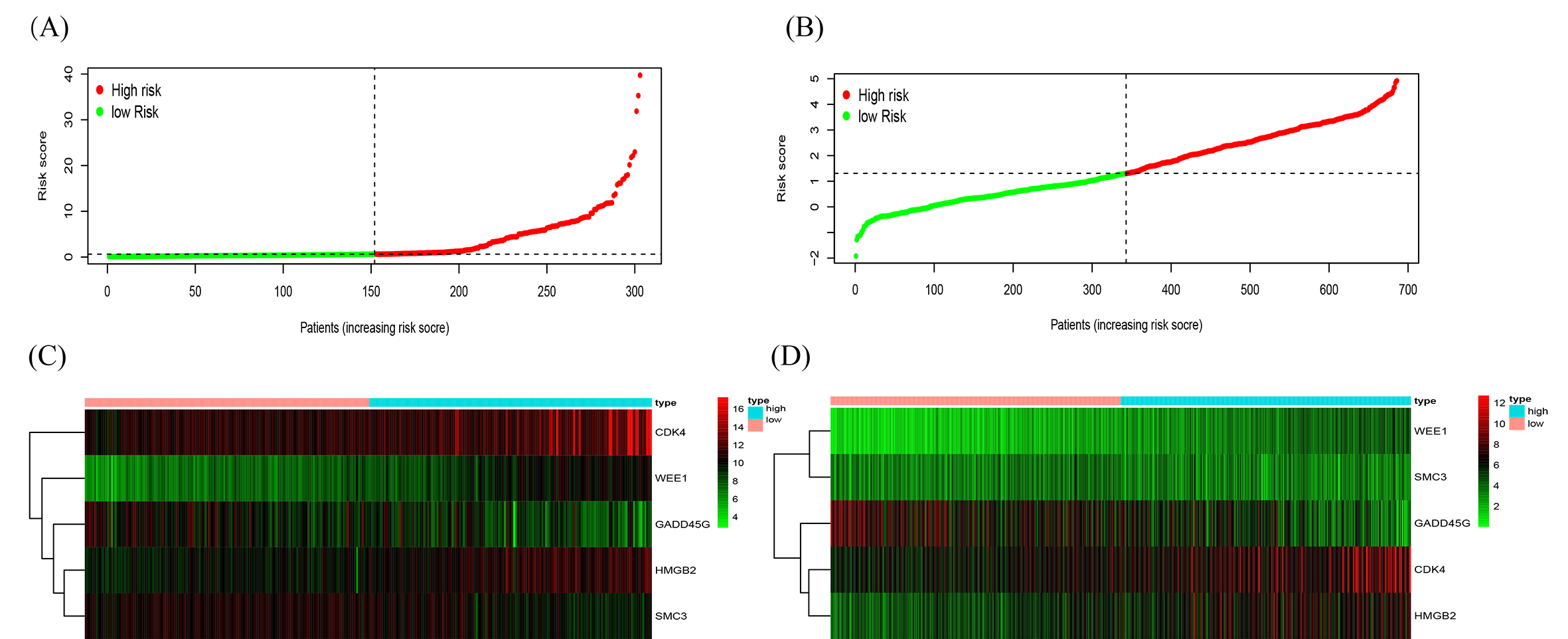

Supplement: Supplementary Figure 4 — TCGA testing and CGGA datasets were used to verify the accuracy of the DDRGs signature. (A, B) Scatterplots illustrating the risk scores for patients with glioma in TCGA testing and CGGA datasets. (C, D) The Heatmap illustrating the expression of the five genes in the high- and low-risk groups. TCGA, The Cancer Genome Atlas; CGGA, Chinese Glioma Genome Atlas; DDRGs, DNA damage repair genes. [file Image_4.tif]

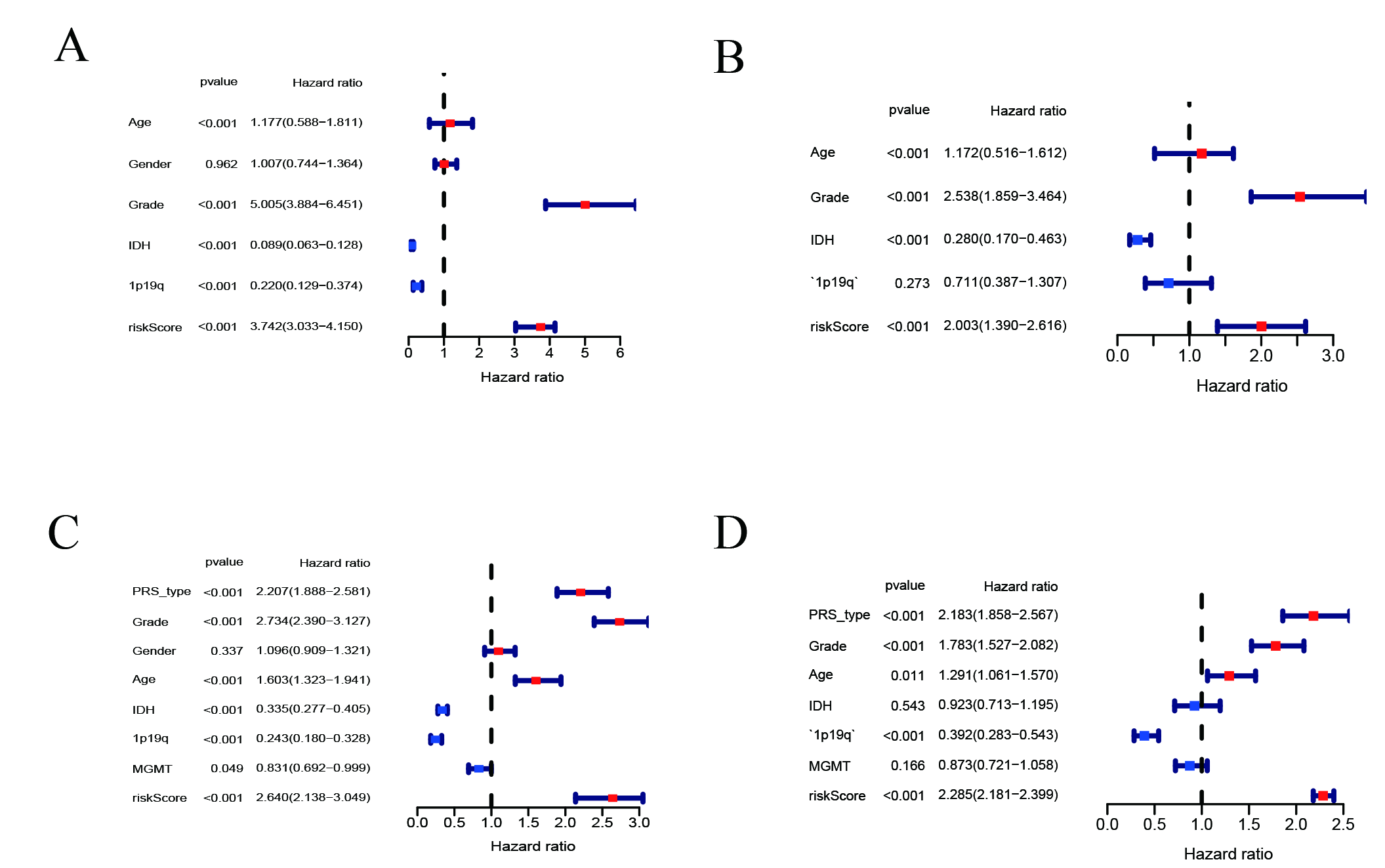

Supplement: Supplementary Figure 5 — Univariate and multivariate Cox analysis of prognostic risk scores for gliomas. Univariate Cox regression analysis. Forest plot of associations between risk factors and the survival of gliomas in the TCGA (A), and CGGA (B). Multiple Cox regression analysis. The DDRGs signature is an independent predictor of gliomas in the TCGA (C), and CGGA (D). TCGA, The Cancer Genome Atlas; CGGA, Chinese Glioma Genome Atlas. [file Image_5.tif]

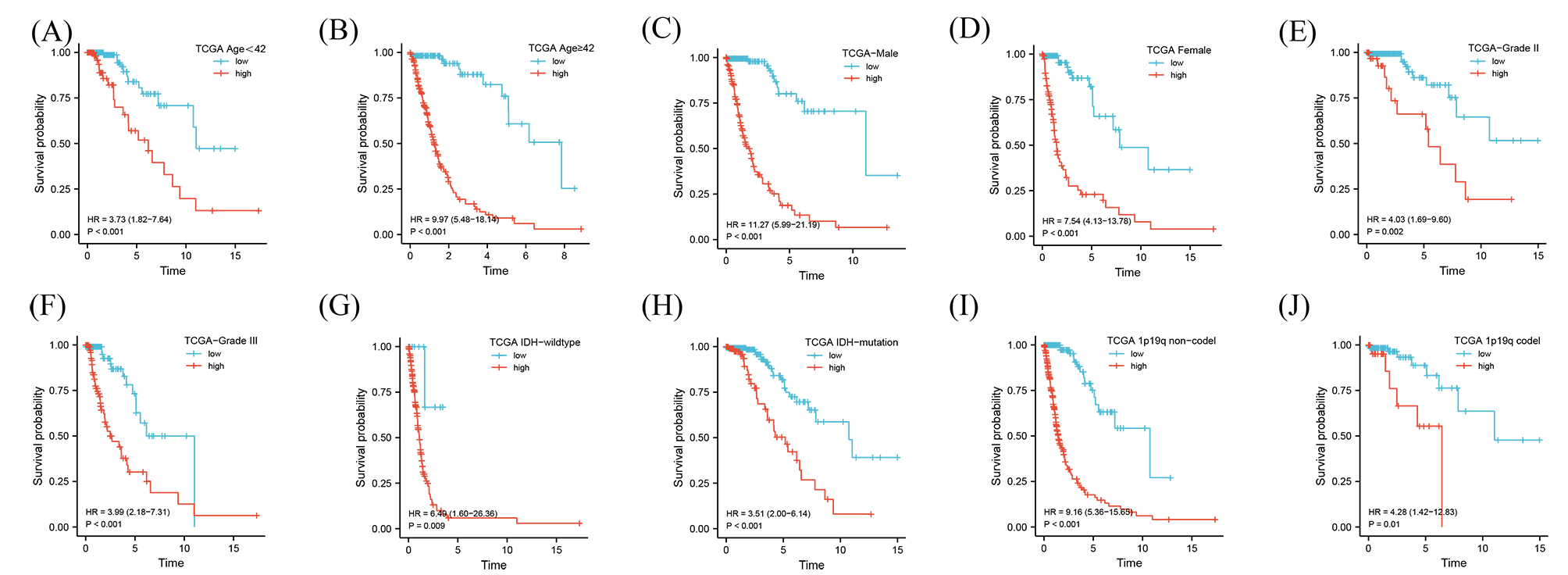

Supplement: Supplementary Figure 6 — Prediction of outcome of the DDRGs signature in stratified patients in TCGA dataset. Survival analysis of the signature in patients stratified by age (A, B), gender (C, D), grade (E, F), IDH (G, H), and 1p19q status (I, J). DDRGs, DNA damage repair genes; TCGA, The Cancer Genome Atlas; IDH, isocitrate dehydrogenase. [file Image_6.tif]

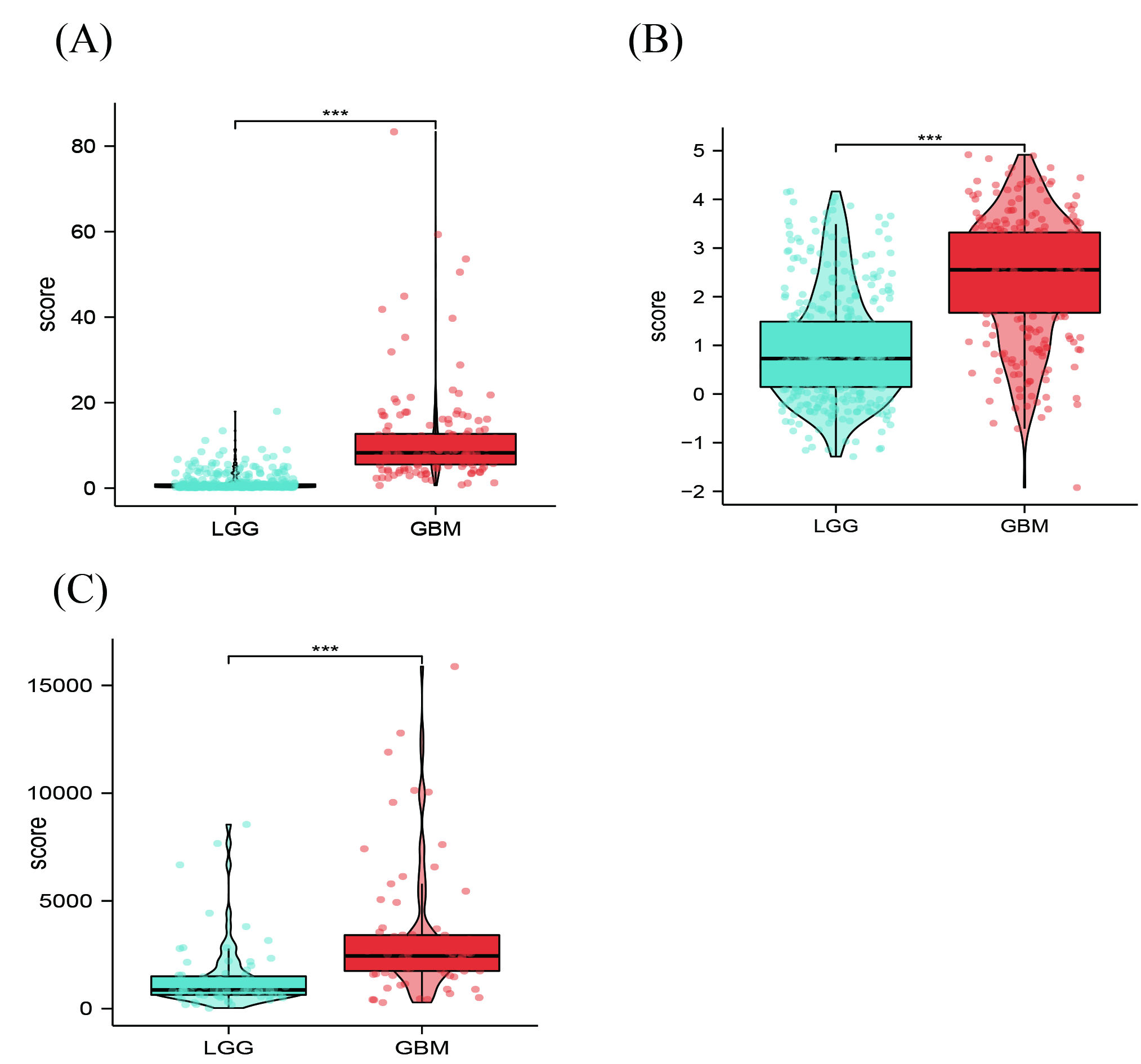

Supplement: Supplementary Figure 7 — Comparison of risk scores in GBM and LGG in the TCGA (A), CGGA (B), and GSE4290 (C). TCGA, The Cancer Genome Atlas; CGGA, Chinese Glioma Genome Atlas. [file Image_7.tif]

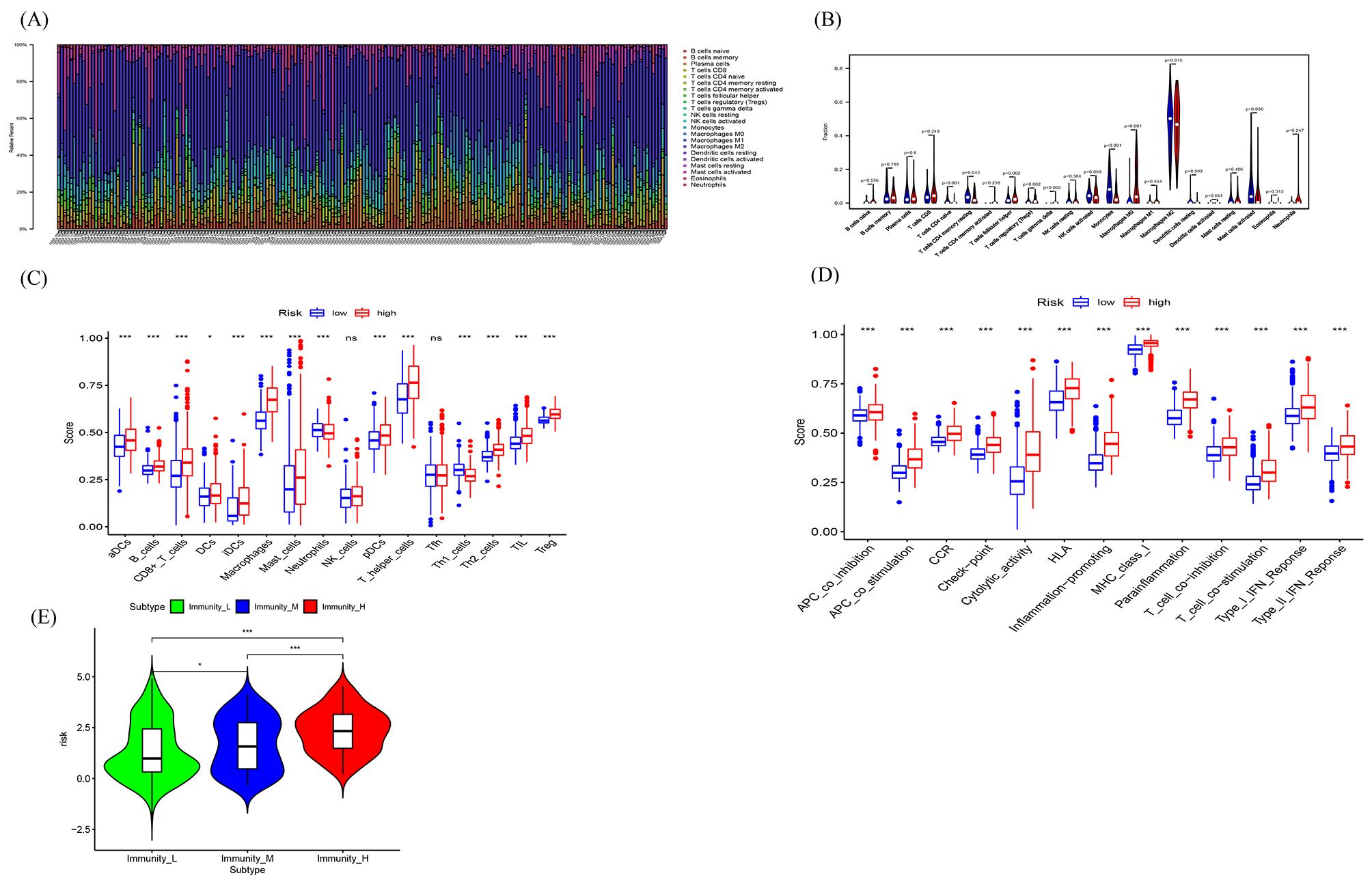

Supplement: Supplementary Figure 8 — Relationship between the DDRGs signature and immune infiltrating cells in the tumor microenvironment. (A) CIBERSORT was used to analyze the types and proportion of infiltrating immune cells in the tumor microenvironment of patients with glioma. (B) Differences in immune infiltrating cells between high- and low-risk groups determined by CIBERSORT. (C, D) Differences in immune infiltrating cells and immune related pathways between high- and low-risk groups determined by ssGSEA. (E) Violin diagram showing the relationship between immune grouping and risk score. DDRGs, DNA damage repair genes; ssGSEA, single-sample gene set enrichment analysis. [file Image_8.tif]
